# Health budget cuts will be paid for by the most vulnerable

**DOI:** 10.4102/safp.v66i1.5934

**Published:** 2024-02-29

**Authors:** Jenny Nash, Andrew J. Ross, Mergan Naidoo, Tasleem Ras, Hanneke Brits, Sheena Mathew

**Affiliations:** 1 Eastern Cape Department of Health, Amathole District, Eastern Cape, South Africa; 2 South African Academy of Family Physicians, Durbanville, South Africa; 3 Department of Family Medicine, Faculty of Medicine, University of KwaZulu-Natal, Durban, South Africa; 4 Department of Family, Community and Emergency Care, Division of Family Medicine, University of Cape Town, Cape Town, South Africa; 5 Department of Family Medicine, Faculty of Health Sciences, University of the Free State, Bloemfontein, South Africa; 6 Private Practice, Blouberg, South Africa

The recent National Treasury announcement in late 2023 that current operating budgets would be slashed to maintain fiscal balance nationally became a reality in health facilities in January 2024. Essential emergency services have been unable to fill medical posts that became vacant on 31 December 2023. The moratorium on new appointments means registrar posts to train medical specialists cannot be filled. From the perspective of primary and district services in rural and urban areas, these consequences span vulnerable communities, overworked and under-resourced health services, junior frontline health workers, and the medical professions’ popular reputation as a meaningful, well-remunerated, and stable source of employment.

Rural and urban communities that use state-funded primary health care clinics and district hospitals are primarily poor and working class, with among the highest rates of unemployment in our society. Ironically, the budget cuts will hit this extremely vulnerable sector of South African society the hardest. Already, many of these facilities do not meet the minimum Office for Health Standard Compliance (OHSC) standards: a prerequisite for national health insurance (NHI). One of the cost-saving measures likely to be implemented is the closure of after-hour services within communities, with all acute and emergency services being provided by the closest hospitals only. This means that those who become sick after-hours must pay for a private vehicle, with costs ranging between R250.00 and R1000.00 per trip. Health economists call this a ‘catastrophic’ health cost, which would be paid from money assigned to food, rental, or school budgets, or worse, sourced from a loan shark. The closure of hospital beds and the loss of locums will further restrict health services, which is likely to specifically affect mental health services and elective surgery.

Recent studies using an internationally validated measuring scale indicated that over 70% of young doctors working in primary care have burnout. The cumulative effects of mental and emotional stress, high workload in substandard facilities, and job instability that are being ushered in by these budget cuts are a sure way to drive these young professionals into the arms of another health system that values them. We hear stories about young healthcare professionals who live away from their families because the areas they work in do not have schools, accepting jobs outside of their careers because they have mouths to feed and debts to pay, and of course, we hear about those who choose to leave the country.

Currently people have access to health services within their communities through the network of primary health care clinics, community health centres and district hospitals. These would include services such as antenatal care, imunisations, medications for chronic diseases and tuberculosis, acute medical problem and trauma. Limiting these services or not appointing competent staff to fill vacant posts will mean that secondary and tertiary services will bear the burden of the excess services, which will have a knock-on effect in many profound ways. By not filling posts, the workload of the remaining staff is increased. In the context of an already overworked staff, this means that stress, burnout, and mental health become problems that may reach epidemic proportions. It is not enough to ask staff to be resilient, to run workshops to build social cohesion between staff, practice mindfulness, do breathing exercises, or journal, while the structural problems that have generated their distress worsen.

The final question that we, as primary care health educators and leaders pose is specifically related to the future of the medical profession in this country. We are still far off the World Health Organization’s doctor : population ratio targets. We urgently need to recruit, graduate, and employ more doctors to serve our most vulnerable populations. However, current evidence suggests that less than 20% of our medical workforce is employed to serve approximately 75% – 80% of our population. With budget cuts, this will be getting worse. How do we convince our brightest young people that medicine is still a viable option when competing with computer science, financial and engineering studies that guarantee a higher income, brighter employment prospects, and arguably (if one believes the data) a better quality of life? Without exaggeration, in South Africa, medicine is in an existential crisis. This budget crisis is forcing us to our knees, the effects of which we may only see in the next decade or so.

We argue that our staff, our services, our communities, and the very existence of the medical profession are at risk of significant and long-lasting damage. Is this a justifiable price to pay for this attempt at balancing the national fiscus? Undoubtedly, other social, service, and educational sectors have been similarly affected, so the damage is not limited to health alone. We call on our provincial and national officials and their political principals to act in the best interests of the most vulnerable in society. Recouping money lost to corruption and mismanagement would easily fund the fiscal shortfall. However, it seems that the voiceless are being made to pay the price on behalf of those in positions of power.

